# Multiplex genetic cancer testing identifies pathogenic mutations in *TP53* and *CDH1* in a patient with bilateral breast and endometrial adenocarcinoma

**DOI:** 10.1186/1471-2350-14-129

**Published:** 2013-12-29

**Authors:** Ellen Heitzer, Sigurd Lax, Ingrid Lafer, Stephanie M Müller, Gunda Pristauz, Peter Ulz, Stephan Jahn, Christoph Högenauer, Edgar Petru, Michael R Speicher, Jochen B Geigl

**Affiliations:** 1 Institute of Human Genetics, Medical University of Graz, Harrachgasse 21/8, A-8010 Graz, Austria; 2 Department of Pathology, General Hospital Graz West, Goestingerstrasse 22, A-8020 Graz, Austria; 3 Department of Obstetrics and Gynecology, Medical University of Graz, Auenbruggerplatz 14, A-8036 Graz, Austria; 4 Institute of Pathology, Medical University of Graz, Auenbruggerplatz 25, A-8036 Graz, Austria; 5 Division of Gastroenterology and Hepatology, Medical University of Graz, Auenbruggerplatz 15, A-8036 Graz, Austria

**Keywords:** Multiplex genetic testing, Cancer susceptibility, TP53, CDH1, Next generation sequencing, NGS

## Abstract

**Background:**

Germline genetic testing for familial cancer syndromes is usually performed serially for the most likely genetic causes. In recent years the way genetic testing carried out has changed, as next generation sequencing now allows the simultaneous testing of multiple susceptibility genes at low costs.

**Case presentation:**

Here, we present a female with bilateral breast cancer and endometrial adenocarcinoma. After simultaneous sequencing of 150 genes (890 kb) associated with hereditary cancer we identified pathogenic mutations in two high-penetrance genes, i.e. *TP53* and *CDH1* that would most likely not have been elucidated by serial screening of candidate genes.

**Conclusion:**

As the two mutated genes are located on different chromosomes and cause different cancer syndromes these findings had a tremendous impact not only on genetic counseling of the index patient and her family but also on subsequent surveillance strategies.

## Background

Testing for mutations in high-penetrance cancer predisposition genes, such as *BRCA1* or *BRCA2*, has evolved to an integral part of cancer care because it provides clear information to patients and their families and established guidelines for surveillance with proven benefit exist (http://www.nccn.org). Germline genetic testing is usually performed serially for the most likely genetic causes [1,2]. As the genetic architecture of cancer predisposition is often complex, genetic testing panels using next-generation sequencing for hereditary cancers have recently been introduced [3]. Here, we present a female with bilateral breast cancer and endometrial adenocarcinoma, where multiplex genetic testing revealed pathogenic mutations in two high-penetrance genes, i.e. *TP53* and *CDH1*. We describe why traditional serial genetic testing would most likely not have elucidated both mutations, which had a tremendous impact on the index patient and her family.

## Methods

The patient and her family received genetic counseling and written informed consent was obtained. BRCA mutation testing using genomic DNA extracted from peripheral blood leukocytes (PBL) was performed. As no mutation was identified, the index patient was included in a study to evaluate a multiplex genetic testing panel for individuals with cancer.

To this end a total of 150 genes corresponding to 890 kb (Additional file 1: Figure S1), which are associated with hereditary cancer or with frequent somatic mutations according to the COSMIC (http://www.sanger.ac.uk/genetics/CGP/cosmic/) and Cancer Gene Census (http://www.sanger.ac.uk/genetics/CGP/Census/) were enriched using a SeqCap EZ Choice Library (Roche Nimblegen, Madison, WI, USA), following the manufacturer’s instructions. Sequencing was performed on the Roche 454FLX platform using the Lib-L protocol (emPCR Method Manual – Lib-L LV).

For cDNA analyses we transcribed leukocyte derived mRNA into cDNA.

## Case presentation

**Figure 1:**
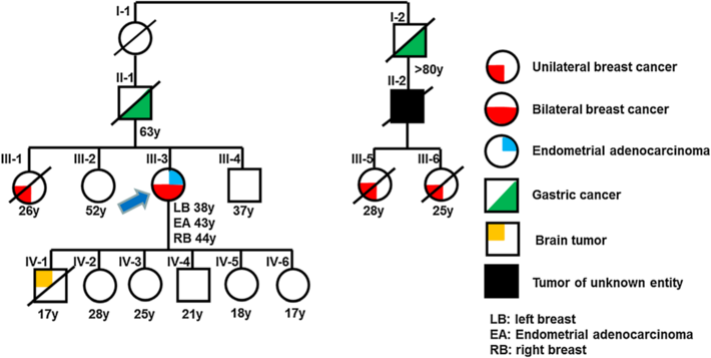
**Pedigree of a family with mutations in both *TP53 *and *CDH1 *showing inheritance of different types of tumors.** Cancer types are marked in different colours. Age in years [y] at diagnosis or current age is indicated below the symbols. Blue arrow indicates the index patient.

The index patient was a 46-year old female (Figure 1; III-3) of Afghan origin. At 38 years of age she was diagnosed with a left sided ductal-invasive breast cancer. At 43 years of age a FIGO Ia endometrial adenocarcinoma was diagnosed, one year later a contralateral, i.e. right sided, invasive breast cancer was detected. She had 4 daughters and two sons (Figure 1; IV-1 to IV-6). One of the sons (Figure 1; IV-1) died from a brain tumor at the age of 17 years. The other children (IV-2 to IV-6) were healthy.

One sister (Figure 1; III-1) of our index patient died at the age of 28 years of breast cancer, diagnosed at 26 years of age, whereas two other siblings (Figure 1; III-2, III-4) were healthy. The mother of our index patient died at the age of 53 years of a myocardial infarction (not shown in the pedigree). On the maternal side there were no cases of known malignant diseases. The family members from the paternal side were all diagnosed and treated in Afghanistan and we were not able to retrieve written documentation. According to the relatives, the father (Figure 1; II-1) died with 63 years of age of gastric cancer. His uncle (Figure 1; I-2) was also diagnosed with gastric cancer at “high age” (>80 years), the son (Figure 1; II-2) of this uncle died of an unknown malignant disease and his two grand-daughters (Figure 1; III-5, III-6) both died at young ages of breast cancer.

The history allowed the differential diagnosis of three autosomal dominantly inherited cancer predisposition syndromes. First, the familial breast-ovarian cancer either due to heterozygous germline mutations in *BRCA1* (OMIM #604370) or *BRCA2* (OMIM #612555), because the index patient and her sister had early-age-onset (≤50 years) breast cancer and the index patient bilateral disease [4]. Second, Li-Fraumeni syndrome (LFS, OMIM #151623) caused by germline mutations of *TP53*, which presents with a variety of tumor types, most notably sarcomas, breast cancer, adrenal cortical carcinoma, and brain tumors [5]. Breast cancer is the most common tumor in women with LFS [6]. *TP53* genetic testing could have been considered according to the Chompret criteria [7,8] because both the index patient and one first-degree relative, i.e. her son, had cancers from the LFS tumor spectrum (breast, brain) before 46 years of age. Third, hereditary diffuse gastric cancer (HDGC, OMIM #137215) characterized by diffuse-type gastric cancer and an elevated risk of lobular breast cancer, which is caused by germline mutations of *CDH1* coding for E-cadherin [9]. However, the current criteria for *CDH1* genetic testing [10] require histopathological confirmation of diffuse gastric cancer in at least one family member, which we did not have, and both gastric cancer cases were not diagnosed below the age of 50 years.

As families who have a predominance of premenopausal breast cancer are more likely to have mutations in *BRCA1* or *BRCA2* than in *TP53*[11], we started our diagnostic work-up with Sanger sequencing and MLPA (Multiplex Ligation-dependent Probe Amplification) of the two breast cancer genes. However, this did not reveal disease-associated mutations.

We next offered the index patient participation in a study with the aim to evaluate a multiplex genetic testing panel for individuals with cancer. This study was approved by the Ethics Committee of the Medical University of Graz. After extensive counseling and obtaining of a written informed consent, we performed targeted resequencing of 150 genes (890 kb) associated with hereditary cancer or with frequent somatic mutations according to the COSMIC (http://www.sanger.ac.uk/genetics/CGP/cosmic/) and Cancer Gene Census (http://www.sanger.ac.uk/genetics/CGP/Census/) databases. SNV calling identified a total of 446 variants. These variants were filtered first for the non-synonymous, splice acceptor-site and donor-site and insertions/deletions mutation and then against available public databases (dbSNP132, 1000 Genome variants databases). After this variant prioritization we identified heterozygous germline mutations in two high-penetrance genes, i.e. *TP53* (c.673-1 G > A) and *CDH1* (c.892 G > A, p.A298T), and one mutation in *NUP214* (c.2160A > C, p.L720F) which we confirmed by Sanger sequencing (Additional file 2: Table S1). The *CDH1* mutation was previously reported in a family with three affected members with diffuse gastric cancer. The mutation dramatically decreases the ability of E-cadherin to mediate cell-cell adhesion and to suppress cell invasion and is therefore listed as causative in HGMD [12,13]. The *TP53* mutation had also been previously observed in a LFS family and analysis of the cDNA had demonstrated that this mutation resulted in a variant transcript [14]. We had only leukocyte derived mRNAs available to confirm this; however, no splice variants were detected in the respective cDNAs. Similar observations with leukocyte derived mRNAs were previously reported for other *TP53* splice site mutations [15] and indicate most likely that the resulting transcripts were unstable and subsequently degraded. As we could not repeat these experiments with an immortalized cell line we carefully analyzed available tumor material from our index patient with immunohistochemistry as outlined below. The *NUP214* mutation was not previously reported. To explore the significance of these mutations further, we analyzed them using various prediction programs including SIFT, PolyPhen, and LRT. As expected for the two previously reported pathogenic mutations in *CDH1* and *TP53* these prediction programs indicated structurally damaging effects. In contrast, these programs suggested for the *NUP214* mutation a non-pathogenic effect. Somatic *NUP214* mutations were mainly reported in cervix and endometrium carcinoma (http://cancer.sanger.ac.uk/cosmic/gene/analysis?ln=NUP214#dist), whereas germline *NUP214* were to best of our knowledge never reported. Altogether this suggests that the *NUP214* mutation does not contribute to the tumor spectrum in our patient.

**Figure 2:**
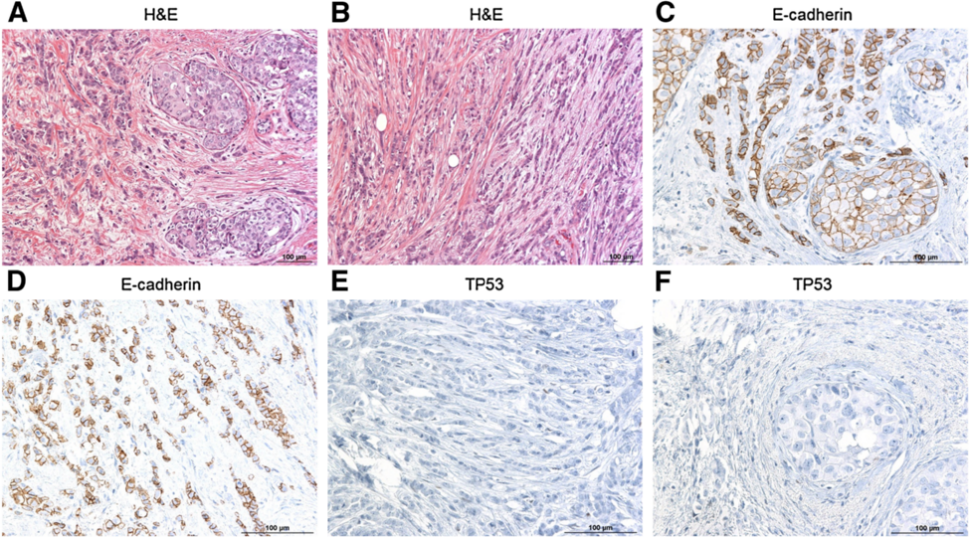
**H& E- and immunostaining of p53 and E-cadherin proteins in different tumor sections. A)** H&E staining of the right-sided breast carcinoma showing an invasive carcinoma, grade 2, with focal lobular features. **B)** H& E staining of the right-sided breast carcinoma showing the lobular component. **C,D)** Immunostaining for E-cadherin. Positive cells (brownish) are present at the membrane but in a clearly lower portion within the component with lobular features. **E,F)** Immunostaining for p53 was negative for both, the invasive and the intraductal carcinoma.

Histology of the left-sided breast carcinoma was not available, since surgery had been performed in Pakistan. All sections of the right-sided breast carcinoma were reviewed and histologically revealed an invasive carcinoma of no special type, grade 2, with focal lobular features (mixed invasive carcinoma) and an associated minor grade 2 intraductal component (Figure 2A). The lobular component was composed of small cells with monomorphic nuclei, arranged in single cell files (Figure 2B). Immunohistochemistry showed membrane staining for E-cadherin, which was less intense within the component with lobular features (Figure 2C-D). TP53 expression was absent in both the invasive and the intraductal carcinoma (Figure 2E-F), indicating that the spliced mutant protein is not expressed. The endometrial adenocarcinoma was of endometrioid type with secretory changes and showed immunoreactivity for E-cadherin, but a flat negative immunoreactive pattern for TP53.

## Conclusion

**Figure 3:**
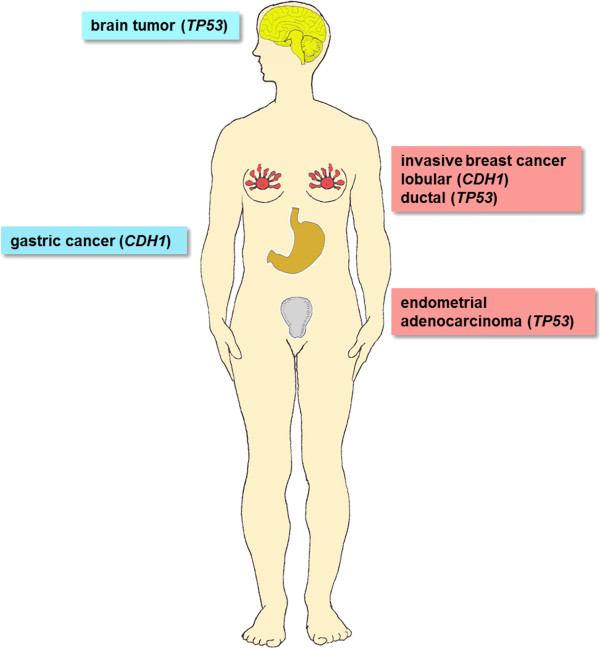
**Different organs are affected with cancer in one family.** Left: tumors observed in males; right: tumors in females; the most likely causative gene is indicated for each tumor.

Our case represents a diagnostic challenge: In the family several different organs are involved, i.e. breast, stomach, uterus, and brain (Figure 3). As several family members were diagnosed and treated abroad, written documentation was incomplete. With traditional serial genetic diagnostics, *TP53* testing would have been the most likely next step after *BRCA1*/*BRCA2* sequencing. Although an uncommon manifestation of LFS, gastric cancer has been suggested to be a component of the LFS tumor spectrum [16] so that all tumors in this family could have been attributed to the *TP53* germline mutation without apparent need for further sequencing of other genes. However, the correct identification of germline mutations is important because LFS and HDGC require intensive and different clinical management.

The lifetime risk of developing cancer is estimated at be as high as 73% for male and 93% for female germline *TP53* mutation carriers [17]. While in the past LFS screening programs often lacked effectiveness a recent novel surveillance protocol using non-invasive biochemical and imaging modalities enabled the presymptomatic detection of malignancies in *TP53* mutation carriers and prolonged survival [18]. Such a surveillance strategy offers new management options and may change attitudes towards genetic *TP53* screening.

Carriers of *CDH1* germline mutations have a cumulative gastric cancer risk, before age 75, of 40–67% for men and 63–83% for women and a risk for lobular breast cancer of 39–52% [19]. Total prophylactic gastrectomy is the only reliable intervention for carriers of pathogenic mutations [9] and was discussed with our patient, illustrating how multiplex genetic testing can change clinical management.

**Figure 4:**
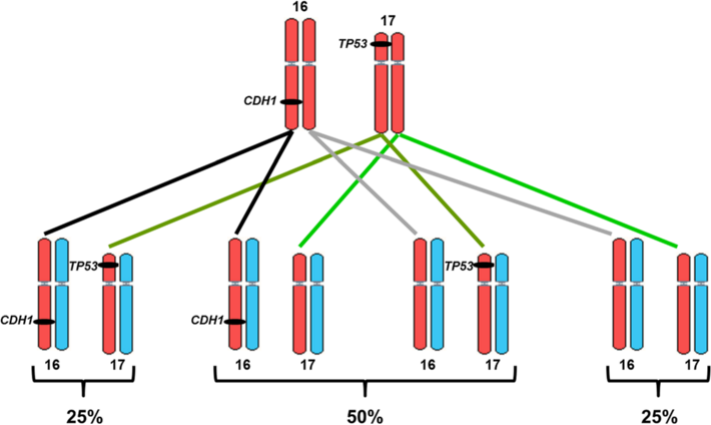
**Possible segregation patterns of the two mutated genes that are located on different chromosomes.** red: maternal; blue: paternal chromosomes.

Furthermore, this situation presents a challenge for genetic counseling as the two mutated genes are located on different chromosomes, i.e. *CDH1* on chromosome 16q22.1 and *TP53* on 17p13.1. Each mutation is inherited with a 50% chance. There is only a 25% probability that a child will inherit no mutation, a 50% chance to inherit one of the two mutated genes and a 25% chance to inherit both (Figure 4). It is very likely that the son, who died at 17 years of age (IV-1 in Figure 1) had at least inherited the mutated *TP53*, as LFS-related brain tumors can occur in either childhood or adulthood with a median age of onset of 16 years [6]. The 28 and 25 year old daughters (IV-2, IV-3) live in Afghanistan and were not available for genetic testing. We identified the *CDH1* but not the *TP53* mutation in the second son (IV-4), whereas no mutation was found in the two younger daughters (IV-5, IV-6).

In summary, we demonstrate a case, where multiplex genetic testing enabled us to establish a diagnosis, which would likely have been missed with serial testing. However, the increased risk for detection of variants of unknown significance represents a concern of multiplex genetic panels [3]. An example is the *NUP214* mutation, which we observed in the index patient and which very likely did not increase tumor susceptibility. To address this issue we now designed gene panels that only include high penetrance gene with clear implications to hereditary tumor syndromes. The growing awareness about the importance of identifying mutation carriers was recently reflected in recommendations released by the American College of Medical Genetics and Genomics [20]. These guidelines state that anyone whose genome is sequenced for any medical reason should automatically learn -without further consent- about mutations in the cancer-predisposition genes for which risk reducing or live saving measures exist. This proposed shift in handling genetic data and the complexity of hereditary multigene cancer panels as illustrated in this report represent novel challenges for cancer genetics professionals.

## Consent

Written informed consent was obtained from the patient for publication of this Case report and any accompanying images. A copy of the written consent is available for review by the Editor of this journal.

## Competing interests

The authors indicated no potential conflicts of interest.

## Authors’ contributions

EH: Project planning and experimental design. Sequencing, next-generation sequencing and data analysis and drafted the manuscript. Manuscript writing, SL: Review of histology IL: Review of clinical data. GP: Review of clinical data. PU: Sequencing, next-generation sequencing and data analysis. SJ: Review of histology. CH: Review of clinical data. EP: Review of clinical data. MRS: Project planning and experimental design, Manuscript writing. JBG: Project planning and experimental designs Review of clinical data, Manuscript writing. All authors read and approved the final manuscript.

## Pre-publication history

The pre-publication history for this paper can be accessed here:

http://www.biomedcentral.com/1471-2350/14/129/prepub

## Supplementary Material

Additional file 1: Figure S1 Electropherogramm and IGV alignment showing mutations in TP53 and CDH1. Click here for file

Additional file 2: Table S1 List of genes that were enriched for sequencing. Click here for file
