# Supplementary figures and images for: Multiplex genetic cancer testing identifies pathogenic mutations in TP53 and CDH1 in a patient with bilateral breast and endometrial adenocarcinoma
Source: BMC Med Genet. 2013 Dec 29;14:129. doi: 10.1186/1471-2350-14-129 (PMC3913615; doi:10.1186/1471-2350-14-129)

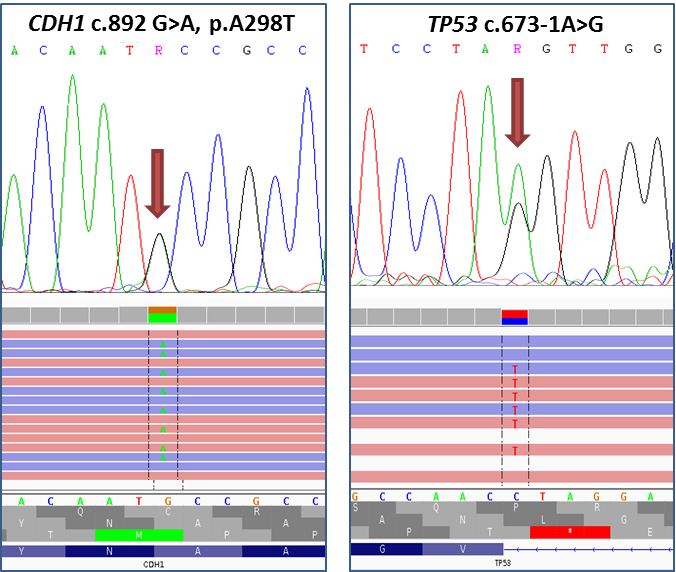

Supplement: Additional file 1: Figure S1 — Electropherogramm and IGV alignment showing mutations in TP53 and CDH1. [file 1471-2350-14-129-S1.tiff]
